# Short-term amino acid, clinicopathologic, and echocardiographic findings in healthy dogs fed a commercial plant-based diet

**DOI:** 10.1371/journal.pone.0258044

**Published:** 2021-10-12

**Authors:** Sarah M. Cavanaugh, Ryan P. Cavanaugh, Gregory E. Gilbert, Elena L. Leavitt, Jennifer K. Ketzis, Aline B. Vieira

**Affiliations:** 1 Department of Clinical Sciences, School of Veterinary Medicine, Ross University, West Farm, Basseterre, St. Kitts; 2 Adtalem Global Education, Downers Grove, Illinois, United States of America; 3 Center for Teaching and Learning, School of Medicine, Ross University, Roseau, Dominica; 4 Department of Biomedical Sciences, School of Veterinary Medicine, Ross University, West Farm, Basseterre, St. Kitts; University of Illinois, UNITED STATES

## Abstract

Consumer demand for commercially prepared plant-based (PB) dog food is increasing, but studies evaluating the short- or long-term effects of PB diets on canine health are lacking. The objective of this study was to assess the short-term amino acid (AA), clinicopathologic, and echocardiographic findings in 34 client-owned dogs fed a commercial extruded plant-based diet (PBD) in which pea protein was the primary protein source and 4 control dogs fed a commercial extruded traditional diet (TD). Plasma AA and whole blood taurine concentrations were measured in dogs at baseline and after 4 weeks on the PBD or the TD. Hematologic, serum biochemical, and echocardiographic testing were performed at baseline and after 12 weeks on the PBD or the TD. Four dogs in the PBD group did not complete the study. All essential AAs, except methionine, were higher in dogs after 4 weeks on the PBD compared to baseline. Taurine (plasma and whole blood) was also higher after 4 weeks on the PBD compared to baseline. A meaningful difference was detected in whole blood taurine between the PBD group and the control group at 4 weeks (*P* = .026) with the PBD group being higher. Median hematologic and biochemical results for the PBD group were within normal limits at baseline and at 12 weeks. In the PBD group, left ventricular internal diastolic dimension (LVIDd, *P* = < .001) and normalized LVIDd (*P* = .031) were higher 12 weeks post-PBD compared to baseline. There were no meaningful differences in left ventricular internal systolic dimension (LVIDs), normalized LVIDs, or fractional shortening 12 weeks post-PBD. There was no statistical evidence of difference between the 2 groups of dogs for any of the echocardiographic parameters at baseline or at 12 weeks. Essential AA or taurine deficiency was not observed in this cohort of dogs fed a commercial extruded PBD. Additionally, clinically relevant hematologic, serum biochemical and echocardiographic alterations were not detected. Further research is required to determine if long-term static feeding of PB diets can meet and maintain AA and other nutrient targets in dogs.

## Introduction

Diets composed of plant-based (PB) foods and devoid of animal products are receiving growing attention across the food chain [1,2]. Interest in extruded PB diets for pets is suspected to be due, in part, to the body of evidence in people demonstrating several health-promoting effects of PB diets. Specifically, dietary patterns characterized by an abundance of healthful plant ingredients have been associated in people with decreased risk of cardiovascular disease, type 2 diabetes, and some cancers [3–5]. PB dietary patterns have also been associated with reduced circulating levels of inflammatory biomarkers in people [6,7]. Other factors contributing to shifts toward plant-based diets globally include concerns surrounding the welfare of animals used for food production and the impact of animal agriculture on the environment [1,8].

PB ingredients have been used in commercially prepared pet diets since the extrusion process was first introduced into the pet food industry in the 1950s. Certain PB foods (e.g., rice, corn) serve as functional ingredients in the extrusion process in that they influence shape, texture, and expansion of kibble. The nutritive content and digestibility of PB ingredients commonly used in commercial pet foods have been studied extensively [9–13], and it is well-established that PB ingredients can serve as viable sources of energy, dietary fiber, digestible carbohydrates, and a variety of other essential and non-essential nutrients for dogs [2,14]. In recent years, consumer demand for alternative protein sources has prompted researchers to investigate amino acid (AA) digestibility and protein quality of several PB ingredients, such as pulses (e.g., beans, lentils), protein concentrates (e.g., pea protein, potato protein), and soy [15–18]. Using precision-fed rooster assays, Reilly and colleagues demonstrated high digestibility of all the indispensable AAs, except methionine, in selected pulse ingredients and high digestibility of all the indispensable AAs in PB protein concentrates [15,16]. Protein quality, assessed by digestible indispensable amino acid scores (DIAAS), indicated methionine was the first-limiting AA in all the pulse ingredients and in all but one of the protein concentrates tested [15,16]. These findings suggest some PB ingredients are viable protein sources for canine and feline diets and emphasize the importance of inclusion of complementary proteins when formulating diets rich in certain PB ingredients.

Despite recommendations and regulations for nutrient content in the global pet food industry, AA deficiencies are still detected in some dogs consuming commercially prepared diets [19–22]. A well-documented cause of myocardial dysfunction in dogs and cats is a deficiency of the sulfur-containing AA, taurine. Dogs with taurine-deficient cardiomyopathy or other nutritionally-related myocardial dysfunction often begin to show improvement in their echocardiographic parameters 12 weeks after treatment or resolution of the underlying cause, but less is known about the time required for nutritionally-related cardiomyopathy to appear echocardiographically in dogs [21–23]. Unlike cats, dogs can synthesize taurine from dietary methionine and cystine; therefore, taurine supplementation has been thought to be unnecessary in dogs who consume a complete and balanced diet. However, taurine deficiency has been documented in dogs fed commercial diets that have been formulated to meet the requirements of Association of American Feed Control Officials (AAFCO) Dog Food Nutrient Profiles [19–22]. Potential explanations include breed-related differences in minimum methionine requirements [24], which are not accounted for by AAFCO, and non-nutrient factors, such as feeding protocols of pet owners, which directly impact caloric intake and nutritional status of pets. More recently, concerns surrounding a possible association between certain commercial diets and dilated cardiomyopathy (DCM)-like disease in dogs have been accumulating [22,23,25–27], and many of the implicated diets have a nutritional adequacy claim stating the diet has been formulated to meet the requirements of AAFCO Dog Food Nutrient Profiles. The discrepancies between nutritional adequacy claims on pet food labels and the health status of dogs consuming the diets underscore the importance of prospective *in vivo* evaluation of commercially available pet foods.

To date, there are no studies evaluating the effects of a PB diet on any naturally-occurring chronic disease in dogs. Moreover, clinical studies assessing for potential negative effects of commercial extruded PB diets on general and/or cardiac health of dogs are lacking. The objectives of this prospective interventional study were to assess the short-term AA, clinicopathologic, and echocardiographic findings in healthy adult dogs fed a commercial extruded PB diet (PBD) in which pea protein was the primary protein source and to compare the post-PBD values to baseline values and to control dogs fed a commercial extruded traditional diet (TD). We hypothesized circulating AA levels, clinicopathologic, and echocardiographic findings of dogs fed a PBD would be similar to baseline and to healthy controls.

## Materials and methods

This study was conducted under a Ross University School of Veterinary Medicine (RUSVM) Institutional Animal Care and Use Committee (IACUC) approved protocol (18.05.19) with all dog owners providing written consent prior to inclusion of their pet(s) in the study.

### Dogs

Dogs were recruited from the student body of an American Veterinary Medical Association-accredited veterinary school. Dogs had to meet the following criteria to be eligible for study enrollment: 1) ≥ 1 year of age; 2) ≥ 5 kg and ≤40 kg; 3) free of clinical signs suggestive of chronic disease (including obesity; defined as a body condition score of ≥8 using a 9-point scale); 4) consuming a TD diet for at least 4 weeks prior to study start; and 5) dogs and their owners had to be living in the vicinity of the university for the duration of the study (September 2018 –December 2018). A TD was defined as commercially available, ≥ 1 animal-based ingredient (i.e., meat, bone meal, and/or animal by-products), non-prescription, and grain-containing (i.e., containing wheat, corn, rice and/or other grains). TDs had to be formulated to meet the requirements of the AAFCO Dog Food Nutrient Profile for adult maintenance or have successfully completed a feeding trial by use of an AAFCO feeding protocol. In addition to the criteria for dog inclusion, owners had to agree to comply with the observational and record-keeping requirements of the study which included completion of a weekly questionnaire assessing dietary acceptance and clinical signs and procuring weekly body weights on the dogs using a scale at the university.

### Initial screening

A questionnaire was distributed to dog owners to gather baseline data and determine if the preliminary inclusion criteria were met (e.g., age, diet). Signalment and history were collected, and forty-three dogs underwent a physical examination by a licensed veterinarian. Five dogs were excluded at this stage: 1 due to chronic arthritis; 1 due to a suspected abdominal mass; 2 because, after discussion, it was determined that the dogs would not be living in the vicinity of the university for the entire study period; and 1 because the owner was unable to comply with the weekly monitoring requirements set forth by the study. The remaining 38 dogs were deemed healthy based on medical history and physical examination and were, therefore, included in the study. All dogs underwent routine jugular venipuncture for laboratory testing, and 37 dogs had an echocardiogram. One dog was not available for echocardiography prior to the start of the study. Initial laboratory testing included: CBC, serum biochemical profile, plasma amino acid panel, and whole blood taurine concentration. Dogs were fasted for 6–12 hours prior blood collection. If a urine sample was available, urinalysis (UA) was performed. Fecal samples were immediately stored at -80°C to preserve the microbiota for future analyses.

For venipuncture, a trained assistant restrained the dogs in a sitting position with the neck held at a 90-degree angle with the thoracic spine. Firm digital pressure was applied (by the phlebotomist) to the jugular furrow, proximal to the phlebotomy site, and 70% isopropyl alcohol was liberally applied to the anticipated site of needle penetration to cleanse the area and facilitate visualization of the vein. Six mls of whole blood were manually procured from the left or right external jugular vein using a sterile 22 gauge hypodermic needle attached to a 6ml, single use, leur lock syringe. Four mls of blood (2mls/tube) were immediately placed into two sterile lithium-heparin vacutainer tubes for AA analysis (see section below). One ml of blood was placed into a purple top EDTA tube and one ml into heparinized green top tube for CBC and serum biochemical analysis, respectively. The samples were transported to the university clinical pathology laboratory for immediate analysis. Complete blood counts were analyzed using Abaxis Vetscan HM5 (Parsipanny, New Jersey, USA) and biochemistry analyses were performed using Abaxis Vetscan VS2 Chemistry Analyzer (Parsippany, New Jersey, USA). A free-catch urine sample (captured mid-stream into a clean stainless steel bowl during normal micturition) and a fecal sample (retrieved manually with gloved finger during digital rectal exam or voluntarily voided immediately before or after exam) were collected from dogs, if feasible. Urinalysis was performed using IDEXX VetLab UA Analyzer (Westbrook, Maine, USA).

### Diets

The PB study diet was procured from a single commercial manufacturer (V-dog, Inc., San Francisco, California, USA) and a single lot. The food label’s nutritional adequacy claim states the diet is formulated to meet the requirements of AAFCO Dog Food Nutrient Profile for adult maintenance. PB treats, from the same manufacturer as the PB diet and from a single lot, were permitted. Three brands of pet food were represented in the TDs; 30 of 38 dogs (79%) were consuming the same brand of food, and 12 dogs (32%) were consuming the same variety of food. For the TDs, 35 dogs (92%) were fed dry (kibble) food exclusively, two dogs (5%) were fed a combination of dry (kibble) and wet (canned) food, and 1 dog was receiving kibble and boiled chicken with stock. Typical nutritional analyses for crude protein and amino acids and ingredient lists of the PBD and the most frequently fed TD can be found in S1 and S2 Appendices, respectively.

### Study design

A longitudinal, parallel design with a 4 week wash-in period was used for this 12 week study. After baseline samples were collected for analyses, 34 of the 38 qualifying dogs were transitioned to the PBD over 5 days, and 4 dogs remained on their TD throughout the study period to serve as a control group. Dogs were assigned to the control group based on owner preference.

Feeding instructions were provided to owners of the dogs in the PB diet group, and owners were required to feed only the kibble and treats provided. Weight-based feeding instructions (amount and frequency) from the PBD company’s website were provided to owners to emulate a real-life scenario of pets. The daily energy requirement (DER) was calculated for each dog and compared to the company’s feeding recommendations to ensure there were no major discrepancies. To calculate DER, resting energy requirement (RER) was determined (70 x [BW]_kg_^.75^) and multiplied by a factor of 1.6 for neutered dogs or 1.8 for intact dogs [28]. Owners were instructed to initially mix 1/3 of the PB diet with 2/3 of the baseline (traditional) diet and then gradually transition to 100% of the PB diet over 5 days. Following the transition period, dogs in both groups underwent a 2^nd^ physical examination. Four weeks later, all dogs underwent a 3^rd^ physical examination and heparinized blood was collected for repeat AA analyses. At 12 weeks, a physical examination and an echocardiogram were performed, and blood was collected for repeat CBC and serum biochemical profile. A urine sample (voided) for urinalysis was also collected from dogs at the 12-week timepoint, if feasible. A fecal sample (voided or retrieved during digital rectal exam) was collected from dogs at the 1-week, 4-week, and 12-week timepoints, if feasible, and stored at -80°C to preserve the microbiota for future analyses. Dog owners completed weekly questionnaires throughout the 12-week study period. Questionnaires collected data on dogs’ body weight, food acceptance, and clinical signs.

### Amino acid analysis

Dogs were fasted for 6–12 hours prior to each blood collection. Samples were collected at a similar time of day (between 12 pm—6 pm) at all timepoints. Two ml aliquots of heparinized blood from each dog were immediately centrifuged and plasma was separated. One 2ml aliquot was reserved, unaltered, in the lithium-heparin tube for whole blood taurine analysis. Plasma and heparinized whole blood samples were stored overnight at -30°C and shipped the following day on ice packs to the AA Laboratory at the University of California Davis School of Veterinary Medicine. One dog’s sample was insufficient for complete AA analysis. At the AA lab, 6% sulfosalicylic acid (containing 400 nMol/ml norleucine as internal STD for QC) was added to each 200ul plasma sample (1:1) in a 1.5ml Eppendorf centrifuge vial for deproteinization. The mixture was thoroughly homogenized using vertex homogenizer and after resting for 15 minutes the mixture was centrifuged at 14000 rpm for 25 minutes under refrigeration (Eppendorf 5430R, 4°C), and then 25ul of supernatant was filtered through a 0.45 mm syringe drive polytetrafluoroethylene (PTFE) filter and pH adjusted to 2.2 before loading on an automated high-performance liquid chromatography AA analyzer (Biochrom 30, BiochromLtd, Holliston, Massachusetts) for quantification. Biochrom 30 amino acid analyzer uses cation exchange column for separation of amino acids and uses post column ninhydrin colorimetric method for quantitation. Peak responses (area) of 570 nm wavelength are for calibrating concentrations of all amino acids except hydroxy-proline and proline. Peak areas of 440nm wavelength are for calibrating concentrations of hydroxy-proline and proline. Sigma A6407 (acidics and neutrals) and A6282 (basics) were used for calibration. NIST standard reference material 2389a was used as control. Heparinized whole blood samples for taurine analysis were frozen and thawed twice to release all taurine before further processing. 150ul distilled and deionized water was added to 50ul well homogenized blood samples in 1.5ml Eppendorf centrifuge vial followed by adding 200ul of 8% SSA containing internal standard. The remainder of processing procedures for taurine were the same as described above.

### Echocardiography

Echocardiograms were performed and interpreted by a board-certified veterinary cardiologist (SMC) using a commercial ultrasound machine (Xario 100, Toshiba, Canon Medical Systems, USA). Dogs were not sedated. Left ventricular internal diastolic and systolic dimensions (LVIDd and LVIDs, respectively) and FS were obtained using 2D-guided M-mode from the right parasternal long-axis left ventricular outflow view. LVIDd and LVIDs were normalized for body weight (LVIDdN, LVIDsN) according to previously described methods [29]. Dogs were excluded from the study if pre-existing heart disease was identified on their initial echocardiogram.

### Statistical analysis

Data were assessed using normal probability plots, Anderson-Darling, Shapiro-Francia, and Shapiro-Wilk tests for normality. Data that were judged to be not normally distributed were judged to be not normally distributed using all methods. The influence of diet on different variables was calculated using Wilcoxon-Mann-Whitney test or Student’s *t* test for independent groups. The effect of time was evaluated using the Wilcoxon sign test or Student’s *t* test for paired samples for all variables. Data is presented as median, interquartile range and/or total range. Analyses were performed using R v3.6.1 (Vienna, Austria.). An *a priori* alpha level of .05 was specified. Data from the 4 dogs who did not complete the study were excluded from statistical analysis.

## Results

### Dogs

Thirty-four dogs transitioned to the PB diet had a median age of 2.9 years (range 1–8.4 years) and median body weight of 19.5 kg (range 9.1–34.4 kg). The following breeds were represented: mixed (27), Siberian husky (2), pit bull (2), border collie (1), vizsla (1), and pug (1). There were 16 spayed females, 15 castrated males, 1 intact female, and 1 intact male. Dogs in the comparison group had a median age of 3.8 years (range 1.5–4.5 years) and a median weight of 20.8 kg (range 15.2–32.8 kg). All 4 dogs in the comparison were mixed breed. Two were spayed females and 2 were castrated males.

Four dogs in the PB diet group did not complete the study. One dog was removed from the study at the end of week 4 because he was not consuming enough of the diet to maintain his body weight (10% weight loss occurred over 4 weeks). A second dog was removed because her consumption of the PB diet was inconsistent, and, at the end of week 3, she began refusing the diet. One dog was withdrawn from the study in week 4 by the owner due to intermittent gastrointestinal signs (diarrhea, flatulence, decreased appetite, borborygmi), which began 3 days after transitioning to the PB diet. This dog also had a history of dietary indiscretion (e.g., eating debris on walks). The dog underwent a complete physical exam shortly after the onset of the first episodes of diarrhea, and no abnormalities were identified. No additional diagnostic testing was performed; therefore, a definitive cause of the signs was not determined. A fourth dog was removed from the study in week 5 for medical reasons (mast cell tumor, urolithiasis).

Eighty percent of owners of the dogs completing the 12-week study described their dog’s acceptance level of the PB diet as “extreme like” or “like,” while 17% said their dog’s acceptance level was “neutral.” One owner (3%) described her dog’s acceptance level of the PB diet as “dislike” during the first half of the study period and “neutral” during the remainder of the study period. Fourteen of 30 dogs (47%) on the PB diet had a total of 24 events of vomiting or diarrhea during the 12-week study period, and all the events occurred after the diet transition period. Most dogs experienced only a single event, and no dog experienced >2 events. Seven of 24 (29%) events were accompanied by an explanation from the owner (e.g., dietary indiscretion). Two of the comparison dogs (50%) had a total of 3 vomiting or diarrhea events during the study period. Events were mild and self-limiting in both groups, and no dog required diagnostic or therapeutic intervention.

### Amino acids

Twenty-nine AAs were assayed. AAs were assessed for normality and the majority were judged to not be normally distributed. Those that were normally distributed were: alanine, arginine, histidine, and tyrosine. AA results for both groups of dogs are presented (Table 1). In the control group, one essential AA, histidine, was higher at 4 weeks compared to baseline (*P* = .017). In the PB diet group, there was statistical evidence of change in the 10 essential AAs and in 12 of the non-essential AAs. All essential AAs, except methionine, were higher after 4 weeks on the PBD. Seven of the non-essential AA, including taurine (plasma and whole blood), were higher after 4 weeks on the PB diet while 5 non-essential AAs were lower after 4 weeks on the PB diet. There were no differences in AA concentrations between the 2 groups of dogs at baseline. At 4 weeks, there were meaningful differences in 5 of the AAs between the 2 groups; cystathionine and whole blood taurine were higher in the PBD group and leucine, methylhistidine-3, and serine were lower in the PBD group. Established reference intervals were available for 25 of the AA assays [30]. Post-PBD AA values, including those that decreased, were within or above the reference intervals, except glutamine and cystine.

**Table 1:** Plasma amino acid and whole blood taurine concentrations in dogs fed a traditional diet or a PB diet for 4 weeks.

Amino acid (nmol/mL)	Traditional diet group (n = 4)			Plant-based diet group (n = 30)			*P*-value between groups		RI^1^
	Baseline	4 weeks	*P*-value between times	Baseline	4 weeks	*P*-value between times	Baseline	4 weeks	
Alanine	444 (392–466)	525 (434–545)	0.401	477(388–540)	407 (354–568)	0.891	0.687	0.518	320–455
*Arginine*	115 (102–132)	155 (114–187)	0.264	133 (111–148)	150 (136–181)	**0.003**	0.276	0.822	85–123
Asparagine	49 (44–58)	53 (47–57)	0.715	56 (47–63)	67 (56–85)	**0.003**	0.485	0.073	30–49
Aspartic acid	11 (7–14)	14 (12–17)	0.144	11 (9–13)	14 (11–16)	**0.017**	0.817	0.857	6–8
Butyric acid	22 (16–40)	27 (18–31)	0.715	22 (15–37)	20 (13–31)	0.206	1.000	0.588	ND
Citrulline	65 (61–90)	107 (81–123)	0.465	70 (50–90)	70 (61–83)	0.750	0.817	0.117	27–50
Cystathionine	5 (4–6)	6 (5–7)	0.465	6 (5–7)	8 (6–9)	**0.003**	0.336	**0.042**	ND
Cystine*	0.8 (0.6–0.8)	1 (1–1)	0.068	1 (0.54–1.51)	2 (1–3)	**0.005**	0.310	0.056	36–56
Glutamic acid	56 (51–59)	113 (104–124)	0.068	54 (47–60)	106 (99–121)	**<0.001**	0.624	0.738	15–26
Glutamine	778 (715–837)	268 (197–324)	0.068	787 (721–860)	269 (223–331)	**<0.001**	0.817	0.777	417–569
Glycine	235 (223–480)	298 (224–598)	0.273	283 (230–340)	229 (207–274)	**0.002**	0.738	0.131	207–310
*Histidine*	87 (84–89)	100 (98–105)	**0.017**	85 (79–92)	100 (95–104)	**<0.001**	0.747	0.894	60–80
Hydroxyproline	29 (26–173)	52 (16–241)	0.465	26 (19–51)	18 (15–27)	**0.003**	0.336	0.285	44–78
*Isoleucine*	62 (58–65)	84 (70–87)	0.144	64 (57–71)	72 (64–79)	**0.041**	0.588	0.310	40–57
*Leucine*	132 (124–142)	177 (170–181)	0.068	132 (124–157)	137 (126–154)	0.600	0.857	**0.002**	95–134
*Lysine*	143 (117–150)	225 (137–319)	0.273	176 (147–210)	187 (166–235)	0.371	0.064	0.519	94–159
*Methionine*	67 (66–68)	75 (69–85)	0.144	70 (63–80)	66 (55–73)	**0.022**	0.588	0.073	45–65
Methylhistidine1	12 (10–14)	11 (9–17)	0.715	10 (8–15)	9 (7–10)	**0.001**	0.453	0.198	ND
Methylhistidine3	18 (12–25)	13 (11–13)	0.273	14 (11–20)	9 (7–10)	**<0.001**	0.897	**0.042**	ND
Ornithine	11 (10–23)	23 (14–39.00)	0.068	14 (12–18)	16 (15–20)	0.106	0.336	0.553	23–43
*Phenylalanine*	58 (57–63)	77 (63–81)	0.144	68 (61–77)	74 (67–83)	**0.020**	0.117	0.738	39–52
Proline	201 (176–363)	321 (178–535)	0.465	173 (139–218)	183 (161–200)	0.719	0.285	0.131	174–304
Serine	106 (90–161)	167 (144–206)	0.068	132 (112–145)	125 (114–146)	0.959	0.453	**0.022**	87–126
Taurine	117 (102–124)	151 (125–179)	0.068	107 (98–121)	166 (153–198)	**<0.001**	0.817	0.262	60–90
*Threonine*	303 (246–355)	363 (256–421)	0.465	239 (183–297)	269 (235–350)	**0.001**	0.239	0.624	138–211
*Tryptophan*	59 (40–64)	78 (74–82)	0.068	58 (39–76)	78 (58–93)	**0.011**	0.738	1.000	45–68
Tyrosine	444 (392–466)	54 (43–59)	0.401	53 (44–61)	61 (53–68)	**<0.001**	0.064	0.077	30–47
*Valine*	115 (102–132)	214 (177–268)	0.264	178 (160–204)	207 (189–231)	**0.020**	0.519	0.777	130–179
WB Taurine	239 (227–243)	256 (224–278)	0.715	244 (222–279)	298 (272–320)	**<0.001**	0.553	**0.026**	224–304

Essential amino acids are italicized. WB = whole blood. ND = not determined. Values presented as median (first quartile–third quartile). *P*-values < 0.05 are bolded.

*Cystine was low at all time points for all groups likely due to storage loss. ^1^Values presented as first quartile–third quartile from Delaney SJ, Kass PH, Rogers QR, Fascetti AJ. Plasma and whole blood taurine in normal dogs of varying size fed commercially prepared food. *J Anim Physiol a Anim Nutri*. 2003;87:236–244.

### Body weight, BCS, hematology, serum biochemistry, and urinalysis

Baseline and post-diet body weight and select hematologic, serum biochemical, and UA data from PBD dogs and from comparison dogs are summarized (Table 2). At the 12-week visit, 2 of the PB diet dogs did not have urine samples available; therefore, data from 28 dogs were included in the analysis. There were no substantial changes in body weight or BCS over the 12-week study period in either group of dogs, and there were no meaningful differences in body weight or BCS between the PB diet dogs and the control dogs at baseline or at 12 weeks. Further, no evidence of nutritional deficiency (e.g., poor haircoat, muscle loss) was detected on physical examination of the dogs at any of the timepoints (baseline, 1 week, 4 weeks, 12 weeks). Median hematologic and biochemical values for both groups of dogs were within normal limits at baseline and at 12 weeks. In the control group, hemoglobin was lower at 12 weeks compared to baseline (*P* = .003). In the PB diet group, ALP (*P* = .047) and urine pH (*P* = .005) were higher at 12 weeks compared to baseline. There were no meaningful hematologic or biochemical differences between the 2 groups at baseline. At 12 weeks, the urine pH of the PB diet group was higher than the urine pH comparison group (*P* = .022). At 12 weeks, two dogs (7%) on the PB diet had an alkaline phosphatase (ALP) concentration that was above the reference interval and 1 dog (3%) had an alanine aminotransferase (ALT) concentration above the reference interval.

**Table 2:** Body weight and select hematologic and serum biochemical data in dogs at baseline and after being fed a traditional diet or a plant-based diet for 12 weeks.

Variable	Traditional diet group (n = 4)			Plant-based diet group (n = 28)			P-value between groups		RI / unit
	Baseline	12 weeks	*P*-value between times	Baseline	12 weeks	*P*-value between times	Baseline	12 weeks	
Body Weight	20.8 (15.2–38.2)	21.4 (14.3–32.7)	0.465	19.5 (9.1–34.4)	19.1 (9.5–36.8)	0.099	0.690	0.770	kg
Hemoglobin*	18.5 (14.7–19.7)	17.8 (15–19.3)	**0.003**	16.8 (14.5–19.9)	16.1 (13.2–19.1)	0.137	0.609	0.021	12–18 g/dL
PCV*	52 (42–55)	52 (43–55)	0.317	47 (41–57)	48 (40–64)	0.799	0.321	0.209	37–55%
Total Protein	6.7 (6.6–7.5)	6.9 (6.7–7.4)	0.414	6.7 (4.1–9.3)	7.0 (5.7–10.1)	0.207	0.624	0.999	5.4–8.2 g/dL
Albumin*	4.1 (3.9–4.3)	4 (3.6–4.2)	0.059	3.8 (2.5–4.4)	3.7 (3.1–4.5)	0.553	0.075	0.075	2.5–4.4 g/dL
ALP*	38 (9–77)	55(26–81)	0.465	34 (6–142)	45 (17–536)	**0.047**	0.938	0.891	20–150 U/L
ALT	39(22–51)	45 (28–47)	0.273	41 (25–88)	44.5 (27–166)	0.079	0.624	0.527	10–118 U/L
Creatinine	1.2 (1–1.4)	1.1 (1–1.5)	0.705	1.2 (0.8–1.5)	1.1 (0.7–2.0)	0.109	0.999	0.457	0.3–1.4 mg/dl
USG	1.04 (1.01–1.05)	1.04 (1.01–1.05)	0.999	1.043 (1.011–1.060)	1.042 (1.011–1.051)	0.188	0.295	0.617	-
Urine pH	6 (5.0–8.0)	5.8 (5.0–6.5)	0.999	6.5 (5.0–9.0)	7.0 (5.0–9.0)	**0.005**	0.730	**0.022**	5.0–9.0

*P*-values < 0.05 are bolded.

*Indicates hematologic and biochemical parameters assessed in AAFCO Dog Food Feeding Protocols.

Values presented as median and range (min-max).

### Echocardiographic findings

Echocardiographic data for all dogs is summarized (Table 3). There were no substantial changes over the 12-week study period in the control group. In the PBD group, LVIDd was higher 12 weeks post-diet compared to pre-diet (*P* < .001). Normalized LVIDd was also higher post-diet (*P* = .031). There were no meaningful changes in LVIDs (*P* = .138), normalized LVIDs (*P* = .317), or FS (*P* = .411) 12 weeks post-PB diet. There was no statistical evidence of difference between the 2 groups of dogs for any of the echocardiographic parameters at baseline or at 12 weeks.

**Table 3:** Left ventricular echocardiographic findings in dogs receiving a traditional diet or a plant-based diet for 12 weeks.

Parameter (unit)	Traditional diet group (n = 4)			Plant-based diet group (n = 29)			*P*-value between groups		RI^1^
	Baseline	12 weeks	*P*-value between times	Baseline	12 weeks	*P*-value between times	Baseline	12 weeks	
LVIDd (mm)	34.45	34.40	0.295	34.20	35.70	**<0.001**	0.567	0.737	NA
LVIDdN	1.41	1.42	1.000	1.41	1.46	**0.031**	0.852	0.690	1.27–1.85
LVIDs (mm)	21.20	21.10	0.514	19.60	20.10	0.138	0.617	0.845	NA
LVIDsN	0.80	0.82	0.317	0.76	0.77	0.317	0.936	0.439	0.71–1.26
FS (%)	39.05	39.3	0.465	41.00	43.40	0.411	0.576	0.203	30–46

Values presented as median. NA = not applicable. *P*-values < 0.05 are bolded.

^1^Cornell, CC, Kittleson MD, Della Torre P, et al. Allometric scaling of M-Mode cardiac measurements in normal adult dogs. *J Vet Intern Med*. 2004;18:311–321.

## Discussion

In this prospective diet study, several changes were observed in the AA profiles of 30 healthy adult dogs after consuming a commercial extruded PBD for 4 weeks. On the basis of the PBD containing a viable primary protein source (pea protein), we hypothesized AA concentrations in the PBD group would be similar to baseline and the control group, which was not the case in this cohort of client-owned dogs. In addition, after 12 weeks on a PBD, clinicopathologic and echocardiographic testing in the dogs demonstrated changes in some of the variables.

The changes in essential AAs, with 9 of 10 being higher at 4 weeks compared to baseline, may be explained in part, by differences in the nutrient content of the baseline (traditional) diet and the PBD (S1 Appendix). Methionine, for example, which was lower in the PBD group compared to baseline, was lower in the nutrient analysis of the PBD compared to the TD. Taurine also was lower in the PBD nutrient analysis; however, plasma and whole blood taurine were significantly higher in the PBD group compared to baseline. Whole blood taurine was also higher in the PBD group compared to controls. While there are no studies that report taurine or other AA concentrations in dogs fed a PBD to compare our results to, there are studies that have investigated the impact of certain plant ingredients on taurine and AA concentrations in dogs and cats. In a study of healthy dogs fed a purified low protein diet, beet pulp, a natural source of dietary fiber, was shown to decrease protein digestibility and increase bile acid excretion thereby reducing circulating taurine levels [31]. In a different study, dogs fed commercially prepared traditional diets containing whole grain brown rice as the first plant ingredient had significantly lower whole blood taurine concentrations than dogs fed a diet with multiple plant protein sources [30]. Healthy cats fed a purified diet containing a high concentration of rice bran developed critically low plasma and whole blood taurine levels compared to controls [32]. Recently, pulses (e.g., peas, lentils), which are limiting in methionine and abundant in fermentable fiber, have been speculated to be associated with some cases of taurine-deficient or other nutritionally-related cardiomyopathy in dogs [22,27,33]. The PBD used in this study did not contain beet pulp or rice bran, but it did contain peas, pea protein, and brown rice as the first 3 ingredients. In addition, the PBD was supplemented with taurine as was the TD; therefore, it is possible the bioavailability of sulfur-containing AAs in either diet was suboptimal and was offset by taurine supplementation. Nevertheless, our finding of higher whole blood taurine levels in dogs fed a PBD for 4 weeks compared to baseline and to controls suggests diets containing only plant ingredients can be formulated and processed to achieve taurine and other nutrient targets in dogs.

Although clinical evidence of amino acid deficiency was not observed in the dogs in this study, two dispensable AAs, glutamine and cystine, were below the established reference intervals in both groups of dogs at 4 weeks. At baseline, however, glutamine concentrations in both groups were above the reference interval. Being that glutamine is a major source of nitrogen for purine synthesis, glutamine levels can fluctuate widely depending on the nitrogen status of the animal [34]. Thus, the plasma glutamine concentrations reported here may simply reflect normal interday variation. Blood collection, sample preparation, and laboratory analysis were standardized; therefore, errors related to sampling and/or analysis are an unlikely cause of the low glutamine levels but cannot be definitively excluded. Cystine was low at baseline and at 4 weeks in both groups of dogs. Protein-bound cystine is lost when plasma proteins are removed before amino acid analysis unless the sample is deproteinized with 6% sulfosalicylic acid [35]. Our samples were not deproteinized prior to shipment to the AA lab; therefore, the low cystine levels in this study were likely due to storage loss during or after shipment.

On the basis that this was an exploratory study, and each test of AA was considered independent of the other, multiplicity adjustments were not made. Being that AA metabolism is influenced by other AAs, statistical significance of differences in the AAs observed in this study must be interpreted with caution. Further, while changes were seen in several of the AA values, interpreting clinical relevance, particularly in regard to the reference intervals, is challenging. The published reference intervals for AA concentrations in dogs are from a single time point [30], and it is unknown whether meaningful day-to-day variations exist in dogs under normal conditions. Gray and colleagues evaluated the effect of 48-hour fasting on AA status in dogs and found that plasma methionine changed 43.7% from baseline after just 12 hours of fasting yet remained within the established reference interval [36]. Intraday variation in AA levels, in association with exercise and diet, has also been observed in dogs [37]. It is possible the fluctuations observed in the AA concentrations of our subjects represent normal interday variation as seen in other species [38,39]. Long-term, randomized, controlled diet trials with AA concentrations from multiple time points would provide valuable information regarding potential normal AA variations in dogs and assist in interpreting the impact of diet.

Vomiting and diarrhea are common clinical signs of primary gastrointestinal (GI) disease in dogs and often occur secondary to non-GI disease (e.g., renal, hepatic) as well which makes them practical and useful signs for monitoring by owners and caretakers [40,41]. The percentage of dogs on the PB diet who had a vomiting or diarrhea event during the 12-week study period (47%) was similar to the comparison group (50%). While 29% of the events in the PB diet group were accompanied by a logical explanation (e.g., known dietary indiscretion), many of the events did not have an explanation. Because the dogs in this study were client-owned and had no environmental or social restrictions, it is possible that 1 or more of the events had an infectious, allergic, or toxic etiology. Nevertheless, the events were transient and did not require medical intervention. We cannot exclude a PBD-related etiology; however, this is considered less likely since a greater percentage of dogs not on the PBD had vomiting or diarrhea at some point during the study period.

Baseline CBC, serum biochemistry, and UA were performed to confirm the health status of the dogs at the time of study inclusion and to provide data for comparison at the end of the 12-week study period. The authors recognize this level of pre- and post-diet testing may be cost prohibitive in other settings (e.g., clinical practice, pet food feeding trials); therefore, using AAFCO post-diet testing requirements [42] as a guide, an abbreviated panel of parameters was created (Table 2). The 2 dogs on the PBD with elevated ALP at the 12-week assessment had no clinical signs and were not taking any medications. The owner of the dog with elevated ALT at the 12-week assessment noted the dog recently had 2 episodes of urinary incontinence, and the dog had a new dermal mass on physical exam. Of the 2 dogs with elevated ALP, 1 had normal ALP at recheck 4 months later and the dog was still being fed the PBD at that time, which suggests the elevation in ALP was likely not diet-related, and the other dog with elevated ALP was lost to follow-up. The dog with elevated ALT had normal ALT on routine bloodwork 10 months later. The dog was no longer consuming the PBD at the time of recheck ALT; therefore, a diet-related cause of increased ALT cannot be excluded. The increase in urine pH in the PB diet group may be attributed to the lack of animal protein and/or abundance of vegetables in the diet [43]. PB diets in people have been shown to increase urine pH and reduce the risk of certain urinary diseases [44–46], and studies in dogs are needed.

Echocardiography was incorporated into the diagnostic plan of this diet study to help provide a comprehensive health assessment of dogs fed a diet for which there are no prior clinical studies. Evidence of overt left ventricular systolic dysfunction was not observed in dogs fed a PBD for 12 weeks. Although increases in LVIDd and LVIDs reached meaningfully different levels at 12 weeks, the observed changes ranged from 1–12% which is within established interday variation of left ventricular echocardiographic parameters in dogs [47]. To the authors’ knowledge, data on minimum or maximum amount of time for diet-induced myocardial dysfunction to develop in dogs do not exist. In a rat model of a vitamin D deficient diet, echocardiographic evidence of cardiac remodeling was detectable at 20 weeks; however, no earlier timepoints were included in that study [48]. The decision to reassess echocardiograms of dogs in this study at 12 weeks was largely based on knowledge that some dogs suffering from nutritionally-related cardiomyopathy begin to show improvement in their echocardiographic parameters as soon as 12 weeks after treatment or resolution of the underlying cause [21–23]. Early surveillance was also chosen in the interest of animal welfare.

A limitation of this study was lack of a randomized control group. The non-randomized group of 4 control dogs and the pre-diet testing provided valuable insight in some circumstances (e.g., cystine concentrations, clinical signs), but neither replaces the verification of observations provided by a randomized control group. Although most of the dogs were consuming the same brand of TD, only a third of dogs were consuming the same variety of food, and this may have impacted AA and other results. The nutrient content of the PB treats was not determined and treat intake was not standardized, which could have led to increased variability of nutrients and reduced our ability to detect differences among the 2 diet groups. Nutrient content of the diets was provided by manufacturers instead of analysis by the researchers. Dog owners and investigators who examined the dogs were not blinded, which could have resulted in reporting bias. A final limitation was the relatively short study period (12 weeks).

The study reported here demonstrated a cohort of 30 healthy adult and predominantly mixed breed dogs who consumed a commercial PBD for 4 weeks underwent changes to their AA profiles. Some of the changes in essential AAs were attributed to differences in AA content of the PBD compared to the AA content of a TD. Additionally, other dietary factors, such as amount, frequency, and global nutrient composition, may have impacted the AA concentrations of the dogs via effects on protein turnover and de novo AA synthesis. Dogs on the PBD did not develop any essential AA deficiencies or taurine deficiency, rather the PBD dogs had taurine concentrations higher than their pre-PBD values and higher than the whole blood taurine concentrations of the control dogs. Results of clinicopathologic and echocardiographic testing indicated the health of the dogs was maintained and did not deteriorate after 12 weeks on a PBD.

Our study evaluated a single commercial PBD; therefore, the results cannot be extrapolated to all PB diets since composition and nutrient availability vary based on formulation and processing. The study reported here supports the need for prospective *in vivo* studies of animal diets and suggests that client-owned pets may be an alternative to traditional study subjects. It should be emphasized that the findings of this study cannot be applied to other species (e.g., cats) nor should veterinary medical professionals or pet owners speculate about the long-term health effects of a PBD based on the results of this short-term study. Further research is needed to elucidate the potential long-term effects of PB diets on health and disease in dogs.

## Supporting information

S1 Dataset Amino acids. (XLSX)

S2 Dataset Body weight and BCS. (XLSX)

S3 Dataset Clinical pathology. (XLSX)

S4 Dataset Echocardiography. (XLSX)

S1 Appendix Nutrient content for crude protein and amino acids from the typical nutritional analysis* of the plant-based diet and the most frequently fed traditional diet. (DOCX)

S2 Appendix Ingredient list* of the plant-based diet and the most frequently fed traditional diet. (DOCX)

## Abbreviations

ALT: alanine aminotransferase
ALP: alkaline phosphatase
AA: amino acid
AAFCO: Association of American Feed Control Officials
BCS: body condition score
FS: fractional shortening
LVIDd: left ventricular internal diameter in diastole
LVIDs: left ventricular internal diameter in systole
LVIDdN: normalized left ventricular diameter in diastole
LVIDsN: normalized left ventricular diameter in systole
PB: plant-based
RI: reference interval
UA: urinalysis
USG: urine specific gravity
