# Supplementary material for: Short-term amino acid, clinicopathologic, and echocardiographic findings in healthy dogs fed a commercial plant-based diet
Source: PLoS One. 2021 Oct 12;16(10):e0258044. doi: 10.1371/journal.pone.0258044 (PMC8509881; doi:10.1371/journal.pone.0258044)
Supplement: S1 Appendix — (DOCX) [file pone.0258044.s005.docx]

Appendix 1: Nutrient content for crude protein and amino acids from the typical nutritional analysis* of the plant-based diet and the most frequently fed traditional diet.

| **Nutrient (unit)** | **Minimum Concentration on DM Basis** | | | | | | |
| --- | --- | --- | --- | --- | --- | --- | --- |
|  | **Traditional diet** |  | **Plant-based diet** |  | **FEDIAF^1^** |  | **AAFCO^2^** |
| Crude Protein (%) | 24.8 |  | 29.3 |  | 18.0 |  | 18.0 |
| Arginine (%) | 1.19 |  | 1.93 |  | 0.52 |  | 0.51 |
| Histidine (%) | 0.55 |  | 0.64 |  | 0.23 |  | 0.19 |
| Isoleucine (%) | 0.85 |  | 1.32 |  | 0.46 |  | 0.38 |
| Leucine (%) | 2.72 |  | 2.39 |  | 0.82 |  | 0.60 |
| Lysine (%) | 0.99 |  | 2.04 |  | 0.42 |  | 0.63 |
| Methionine (%) | 0.56 |  | 0.52 |  | 0.40 |  | 0.33 |
| Methionine-Cystine (%) | 0.94 |  | 0.88 |  | 0.76 |  | 0.65 |
| Phenylalanine (%) | 1.23 |  | 1.52 |  | 0.54 |  | 0.45 |
| Phenylalanine-Tyrosine (%) | 2.15 |  | 2.43 |  | 0.89 |  | 0.74 |
| Taurine (%) | 1.05 |  | 0.21 |  | 0.52 |  | ND |
| Tryptophan (%) | 0.22 |  | 0.35 |  | 0.17 |  | 0.16 |
| Valine (%) | 1.06 |  | 1.54 |  | 0.59 |  | 0.49 |
| Calculated Energy Density (kcal/kg) | 3657 |  | 3675 |  | 4000 |  | 4000 |

*Provided by the pet food companies.

DM = dry matter; FEDIAF = European pet food industry; AAFCO = Association of American Feed Control Officials; ND = not determined. ^1^FEDIAF Recommended Nutrient Levels for Adult Dogs based on MER of 110 kg/kg^.75^. In: *FEDIAF Nutritional Guidelines September 2020*. Bruxelles, Belgium. ^2^AAFCO Dog Food Nutrient Profile (minimum adult maintenance) based on dry matter. In: *2020 Official Publication.* Champaign, Illinois, USA.
