# Supplementary material for: Short-term amino acid, clinicopathologic, and echocardiographic findings in healthy dogs fed a commercial plant-based diet
Source: PLoS One. 2021 Oct 12;16(10):e0258044. doi: 10.1371/journal.pone.0258044 (PMC8509881; doi:10.1371/journal.pone.0258044)
Supplement: S2 Appendix — (DOCX) [file pone.0258044.s006.docx]

Appendix 2: Ingredient list* of the plant-based diet and the most frequently fed traditional diet.

**Plant-based diet:** Dried Peas, Pea Protein, Brown Rice, Oatmeal, Potato Protein, Sorghum, Canola Oil (preserved with mixed tocopherols) , Natural Flavor, Suncured Alfalfa Meal, Brewers Dried Yeast, Dicalcium Phosphate, Flaxseeds, Millet, Calcium Carbonate, Lentils, Peanut Hearts, Quinoa, Sunflower Chips, Salt, Potassium Chloride, Choline Chloride, Taurine, Dried Carrots, Minerals (Ferrous Sulfate, Zinc Sulfate, Copper Sulfate, Sodium Selenite, Manganese Sulfate, Calcium Iodate), Dl-methionine, Dried Parsley, Vitamins (Vitamin E Supplement, Vitamin A Supplement, Niacin Supplement, D-calcium Pantothenate, Riboflavin Supplement, Vitamin D2 Supplement, Thiamine Mononitrate, Vitamin B12 Supplement, Pyridoxine Hydrochloride, Biotin, Folic Acid), L-Ascorbyl-2-Polyphosphate (A Source Of Vitamin C), Preserved with Citric Acid, Preserved with mixed Tocopherols, Dried Blueberries, Dried Cranberries, Dried Celery, Yucca Schidigera Extract, Dried Lettuce, L-carnitine, Dried Watercress, Dried Spinach, Rosemary Extract.

**Traditional diet:** Chicken, Cracked Pearled Barley, Whole Grain Wheat, Whole Grain Corn, Whole Grain Sorghum, Corn Gluten Meal, Soybean Meal, Chicken Fat, Brewers Rice, Chicken Liver Flavor, Chicken Meal, Dried Beet Pulp, Soybean Oil, Pork Flavor, Lactic Acid, Calcium Carbonate, Flaxseed, Potassium Chloride, Choline Chloride, Iodized Salt, vitamins (Vitamin E Supplement, L-Ascorbyl-2-Polyphosphate [source of Vitamin C], Niacin Supplement, Thiamine Mononitrate, Vitamin A Supplement, Calcium Pantothenate, Riboflavin Supplement, Biotin, Vitamin B12 Supplement, Pyridoxine Hydrochloride, Folic Acid, Vitamin D3 Supplement), Taurine, minerals (Ferrous Sulfate, Zinc Oxide, Copper Sulfate, Manganous Oxide, Calcium Iodate, Sodium Selenite), Oat Fiber, Mixed Tocopherols for freshness, Natural Flavors, Beta-Carotene, Apples, Broccoli, Carrots, Cranberries, Green Peas.

*Obtained from pet food labels.
